# *catena*-Poly[[[triaqua­[3-(4-carb­oxy­phen­oxy)phthalato-κ*O* ^2^]manganese(II)]-μ-4,4′-bipyridine-κ^2^ *N*:*N*′] 4,4′-bipyridine monosolvate dihydrate]

**DOI:** 10.1107/S1600536813000585

**Published:** 2013-01-23

**Authors:** Wei Sun

**Affiliations:** a Department of Chemistry, University of Science and Technology Beijing, Beijing 100083, People’s Republic of China

## Abstract

In the title compound, {[Mn(C_15_H_8_O_7_)(C_10_H_8_N_2_)(H_2_O)_3_]·C_10_H_8_N_2_·2H_2_O}_*n*_, the bridging mode of the coordinating 4,4′-bipyridine ligands leads to the formation of polymeric zigzag chains parallel to [0-11]. The chains are separated by 4,4′-bipyridine and water solvent mol­ecules. Within a chain, the Mn^II^ atom is six-coordinated by two N atoms of the bridging 4,4′-bipyridine ligands, three water O atoms and one carboxyl­ate O atom of a single deprotonated 3-(4-carb­oxy­phen­oxy)phthalic acid ligand. Both coordinating and solvent 4,4′-bipyridine mol­ecules are situated on centres of inversion. An intricate network of O—H⋯O and O—H⋯N hydrogen bonds involving the carb­oxy group, the coordinating water mol­ecules and the two types of solvent mol­ecules leads to the formation of a three-dimensional network.

## Related literature

For applications of metal-organic coordination polymers, see: Leininger *et al.* (2000 ▶). For a related structure, see: Wang *et al.* (2009 ▶). For synthetic details, see: Cai (2011 ▶); Wang *et al.* (2010 ▶).
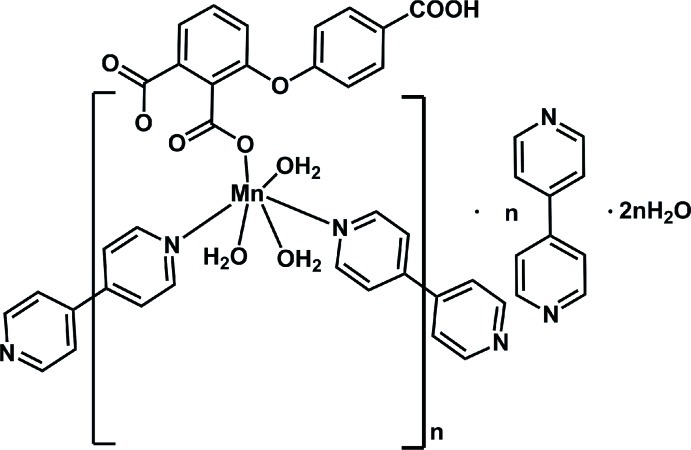


## Experimental

### 

#### Crystal data

[Mn(C_15_H_8_O_7_)(C_10_H_8_N_2_)(H_2_O)_3_]·C_10_H_8_N_2_·2H_2_O
*M* *_r_* = 757.60
Triclinic, 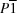

*a* = 10.765 (1) Å
*b* = 11.883 (2) Å
*c* = 14.574 (1) Å
α = 110.275 (3)°
β = 95.028 (1)°
γ = 94.970 (1)°
*V* = 1728.4 (4) Å^3^
*Z* = 2
Mo *K*α radiation
μ = 0.45 mm^−1^
*T* = 293 K
0.18 × 0.14 × 0.10 mm

#### Data collection

Bruker APEXII CCD area-detector diffractometer
Absorption correction: multi-scan (*SADABS*; Bruker, 2004 ▶) *T*_min_ = 0.923, *T*_max_ = 0.956
9338 measured reflections
6673 independent reflections
5695 reflections with *I* > 2σ(*I*)
*R*_int_ = 0.016

#### Refinement

*R*[*F* ^2^ > 2σ(*F* ^2^)] = 0.034
*wR*(*F* ^2^) = 0.095
*S* = 1.07
6673 reflections
469 parameters
18 restraints
H-atom parameters constrained
Δρ_max_ = 0.25 e Å^−3^
Δρ_min_ = −0.28 e Å^−3^

### 

Data collection: *APEX2* (Bruker, 2004 ▶); cell refinement: *SAINT-Plus* (Bruker, 2004 ▶); data reduction: *SAINT-Plus*; program(s) used to solve structure: *SHELXS97* (Sheldrick, 2008 ▶); program(s) used to refine structure: *SHELXL97* (Sheldrick, 2008 ▶); molecular graphics: *XP* in *SHELXTL* (Sheldrick, 2008 ▶); software used to prepare material for publication: *SHELXL97*.

## Supplementary Material

Crystal structure: contains datablock(s) global, I. DOI: 10.1107/S1600536813000585/wm2704sup1.cif

Structure factors: contains datablock(s) I. DOI: 10.1107/S1600536813000585/wm2704Isup2.hkl

Additional supplementary materials:  crystallographic information; 3D view; checkCIF report

## Figures and Tables

**Table 1:** Hydrogen-bond geometry (Å, °)

*D*—H⋯*A*	*D*—H	H⋯*A*	*D*⋯*A*	*D*—H⋯*A*
O7—H7*A*⋯N4^i^	0.94	1.64	2.577 (2)	170
O8—H8*B*⋯N3^ii^	0.90	1.94	2.814 (2)	166
O8—H8*A*⋯O3	0.95	1.76	2.662 (2)	158
O9—H9*A*⋯O2	0.95	1.87	2.8035 (19)	165
O9—H9*B*⋯O2^iii^	0.89	1.77	2.6595 (18)	174
O10—H10*A*⋯O11^iv^	0.99	1.78	2.771 (2)	173
O10—H10*B*⋯O1^iii^	0.82	1.84	2.6545 (19)	174
O11—H11*B*⋯O12	0.88	1.93	2.803 (3)	170
O11—H11*A*⋯O6^v^	0.84	2.02	2.847 (2)	168
O12—H12*A*⋯O3^vi^	0.85	2.14	2.802 (3)	134
O12—H12*B*⋯O2^vi^	0.85	2.45	3.139 (3)	139

Symmetry codes: (i) 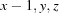; (ii) 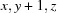; (iii) 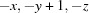; (iv) 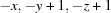; (v) 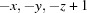; (vi) 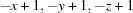.

**Fig. 1.**
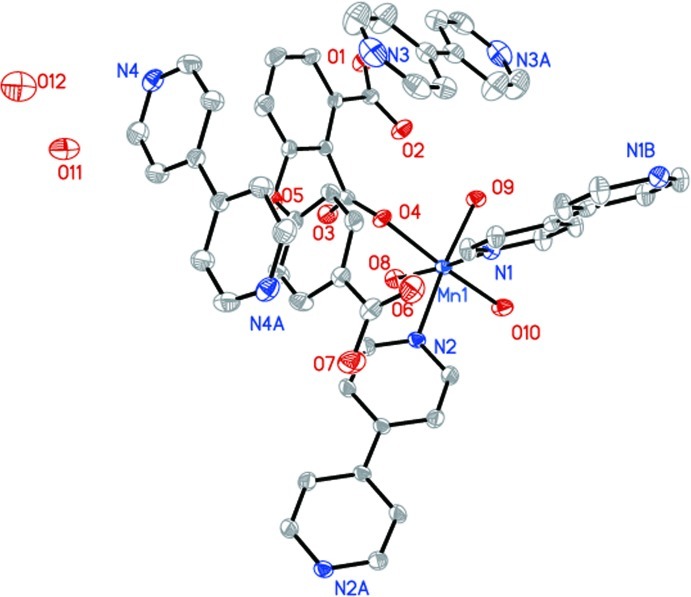
The coordination of MnII in the structure of (I). Displacement ellipsoids are drawn at the 30% probability level. The solvent 4,4'-bipyridine as well as the two solvent water molecules are also shown. All H atoms were omitted for clarity. [Symmetry codes: A) -1-x, -y, -z; B) -1-x, 1-y, 1-z.]

**Fig. 2.**
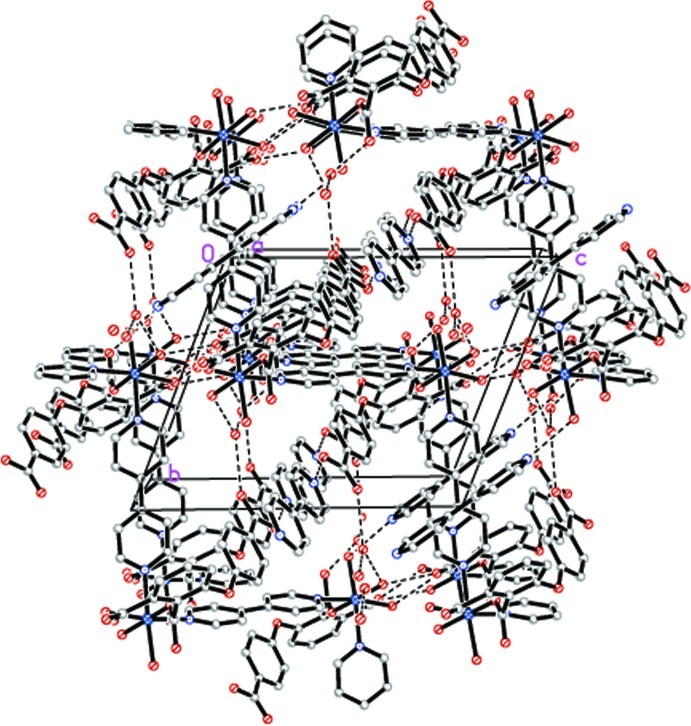
A view of three-dimensional supramolecular structure of (I) resulting from the hydrogen bonding (dashed lines represent donor···acceptor interactions).

## supplementary crystallographic information

### Comment

The design of metal-organic coordination polymers with interesting structures and properties has stimulated the interests of scientists in the field of supramolecular chemistry and crystal engineering over the past few decades (Leininger *et al.*, 2000). Recently, considerable progress has been achieved in the preparation of coordination polymers with desired functionalities. In the present work, the novel coordination polymer, [Mn(C_15_H_8_O_7_)(H_2_O)_3_(C_10_H_8_N_2_)]^.^C_10_H_8_N_2_^.^2H_2_O, (I), has been prepared hydrothermally, and its structure is described here.

The asymmetric unit of compound (I) is composed of a 3-(4-carboxyphenoxy) phthalate ligand (*L*), two halves of two 4,4'-bipyridine ligands, a divalent manganese ion, three coordinating water molecules, two halves of 4,4'-bipyridine solvent molecules and two solvent water molecules. The ligand *L* is in a single deprotonated form. The Mn^II^ atom is octahedrally coordinated by two N atoms of two bridging 4,4'-bipyridine ligands, three water O atoms and one O atom of a carboxylate function of *L* (Fig. 1). The bridging mode of the 4,4'-bipyridine ligands leads to the formation of zig-zag chains extending parallel to [011].

Extensive O—H···O and O—H···N hydrogen bonding between water molecules and the carboxy function as donors and 4,4'-bipyridine molecules, carboxyate groups, and water molecules as acceptors (Table 1) leads to the construction of a three-dimensional supramolecular structure (Fig. 2). The hydrogen-bonding scheme resembles that of a related structure discussed by Wang *et al.* (2009).

### Experimental

Compound (I) was synthesized referring to a procedure given by Cai (2011) and Wang *et al.* (2010). A mixture containing Mn(OAc)_2_^.^4H_2_O (0.049 g, 0.2 mmol), 4,4'-bipyridine (0.031 g, 0.2 mmol), 3-(4-carboxyphenoxy)phthalate (0.030 g, 0.1 mmol), and H_2_O (15 ml) was sealed in a Teflon-lined stainless steel reactor and heated to 393 K. Pale yellow crystals were separated by filtration and dried in air; yield *ca*. 32%.

### Refinement

All C-bound H atoms were placed in geometrically idealized positions and treated as riding on their parent atoms with C—H = 0.93 Å and *U*_iso_ = 1.2U_eq_(C). The H atoms associated with the carboxyl group and the water molecules were clearly discernible from difference maps. They were refined with distance restraints (O—H and H—H) by using the *DFIX* command in SHELXTL, and with *U*_iso_(H) = 1.5U_eq_(O).

### Crystal data

**Table:**

[Mn(C_15_H_8_O_7_)(C_10_H_8_N_2_)(H_2_O)_3_]·C_10_H_8_N_2_·2H_2_O	*Z* = 2
*M**_r_* = 757.60	*F*(000) = 786
Triclinic, *P*1	*D*_x_ = 1.456 Mg m^−^^3^
Hall symbol: -P 1	Mo *K*α radiation, λ = 0.71069 Å
*a* = 10.765 (1) Å	Cell parameters from 4836 reflections
*b* = 11.883 (2) Å	θ = 2.8–27.3°
*c* = 14.574 (1) Å	µ = 0.45 mm^−^^1^
α = 110.275 (3)°	*T* = 293 K
β = 95.028 (1)°	Block, pale yellow
γ = 94.970 (1)°	0.18 × 0.14 × 0.10 mm
*V* = 1728.4 (4) Å^3^	

### Data collection

**Table:**

Bruker APEXII CCD area-detector diffractometer	6673 independent reflections
Radiation source: fine-focus sealed tube	5695 reflections with *I* > 2σ(*I*)
Graphite monochromator	*R*_int_ = 0.016
φ and ω scans	θ_max_ = 26.0°, θ_min_ = 1.8°
Absorption correction: multi-scan (*SADABS*; Bruker, 2004)	*h* = −13→13
*T*_min_ = 0.923, *T*_max_ = 0.956	*k* = −14→11
9338 measured reflections	*l* = −17→17

### Refinement

**Table:**

Refinement on *F*^2^	Primary atom site location: structure-invariant direct methods
Least-squares matrix: full	Secondary atom site location: difference Fourier map
*R*[*F*^2^ > 2σ(*F*^2^)] = 0.034	Hydrogen site location: inferred from neighbouring sites
*wR*(*F*^2^) = 0.095	H-atom parameters constrained
*S* = 1.07	*w* = 1/[σ^2^(*F*_o_^2^) + (0.0504*P*)^2^ + 0.3087*P*] where *P* = (*F*_o_^2^ + 2*F*_c_^2^)/3
6673 reflections	(Δ/σ)_max_ = 0.001
469 parameters	Δρ_max_ = 0.25 e Å^−^^3^
18 restraints	Δρ_min_ = −0.28 e Å^−^^3^

### Special details

**Table:**

Geometry. All e.s.d.'s (except the e.s.d. in the dihedral angle between two l.s. planes) are estimated using the full covariance matrix. The cell e.s.d.'s are taken into account individually in the estimation of e.s.d.'s in distances, angles and torsion angles; correlations between e.s.d.'s in cell parameters are only used when they are defined by crystal symmetry. An approximate (isotropic) treatment of cell e.s.d.'s is used for estimating e.s.d.'s involving l.s. planes.
Refinement. Refinement of *F*^2^ against ALL reflections. The weighted *R*-factor *wR* and goodness of fit *S* are based on *F*^2^, conventional *R*-factors *R* are based on *F*, with *F* set to zero for negative *F*^2^. The threshold expression of *F*^2^ > σ(*F*^2^) is used only for calculating *R*-factors(gt) *etc*. and is not relevant to the choice of reflections for refinement. *R*-factors based on *F*^2^ are statistically about twice as large as those based on *F*, and *R*- factors based on ALL data will be even larger.

### Fractional atomic coordinates and isotropic or equivalent isotropic displacement parameters (Å^2^)

**Table:**

	*x*	*y*	*z*	*U*_iso_*/*U*_eq_	
C1	0.22064 (17)	0.38214 (17)	0.09394 (13)	0.0351 (4)	
C2	0.21923 (16)	0.32151 (16)	0.16976 (12)	0.0306 (4)	
C3	0.30439 (18)	0.23977 (18)	0.16908 (14)	0.0405 (4)	
H3	0.3594	0.2230	0.1217	0.049*	
C4	0.3092 (2)	0.1829 (2)	0.23714 (17)	0.0497 (5)	
H4	0.3654	0.1271	0.2347	0.060*	
C5	0.2290 (2)	0.2101 (2)	0.30932 (16)	0.0460 (5)	
H5	0.2319	0.1736	0.3564	0.055*	
C6	0.14559 (16)	0.29131 (17)	0.31048 (13)	0.0331 (4)	
C7	0.13674 (15)	0.34762 (15)	0.24158 (12)	0.0279 (3)	
C8	0.04632 (16)	0.44143 (16)	0.25351 (12)	0.0297 (4)	
C9	−0.03649 (17)	0.26350 (17)	0.38774 (12)	0.0326 (4)	
C10	−0.10301 (19)	0.31301 (19)	0.46616 (14)	0.0436 (5)	
H10	−0.0692	0.3839	0.5175	0.052*	
C11	−0.2197 (2)	0.25630 (19)	0.46742 (15)	0.0452 (5)	
H11	−0.2649	0.2898	0.5198	0.054*	
C12	−0.27071 (17)	0.14997 (17)	0.39170 (14)	0.0362 (4)	
C13	−0.19944 (18)	0.09888 (18)	0.31669 (14)	0.0389 (4)	
H13	−0.2310	0.0255	0.2673	0.047*	
C14	−0.08209 (18)	0.15488 (18)	0.31369 (13)	0.0390 (4)	
H14	−0.0352	0.1200	0.2629	0.047*	
C15	−0.40006 (18)	0.09199 (19)	0.38805 (16)	0.0414 (5)	
C16	−0.27139 (18)	0.5215 (2)	0.39115 (14)	0.0424 (5)	
H16	−0.1840	0.5327	0.4007	0.051*	
C17	−0.33425 (18)	0.5189 (2)	0.46953 (13)	0.0411 (5)	
H17	−0.2889	0.5278	0.5294	0.049*	
C18	−0.46457 (16)	0.50325 (16)	0.45902 (12)	0.0308 (4)	
C19	−0.52447 (18)	0.4919 (2)	0.36711 (14)	0.0437 (5)	
H19	−0.6118	0.4822	0.3558	0.052*	
C20	−0.45483 (18)	0.4948 (2)	0.29281 (14)	0.0418 (5)	
H20	−0.4977	0.4866	0.2322	0.050*	
C21	−0.33741 (19)	0.22192 (17)	0.15354 (14)	0.0397 (4)	
H21	−0.2945	0.2486	0.2170	0.048*	
C22	−0.40125 (18)	0.10654 (17)	0.11631 (14)	0.0377 (4)	
H22	−0.4018	0.0585	0.1549	0.045*	
C23	−0.46453 (16)	0.06216 (15)	0.02145 (13)	0.0301 (4)	
C24	−0.4605 (2)	0.14042 (18)	−0.03056 (14)	0.0427 (5)	
H24	−0.5011	0.1153	−0.0947	0.051*	
C25	−0.3962 (2)	0.25575 (18)	0.01303 (14)	0.0433 (5)	
H25	−0.3966	0.3069	−0.0229	0.052*	
C26	−0.1322 (3)	−0.1451 (2)	0.10978 (18)	0.0611 (6)	
H26	−0.2113	−0.1668	0.1239	0.073*	
C27	−0.1236 (2)	−0.0724 (2)	0.05370 (17)	0.0540 (5)	
H27	−0.1954	−0.0462	0.0316	0.065*	
C28	−0.0072 (2)	−0.03880 (19)	0.03055 (15)	0.0456 (5)	
C29	0.0937 (3)	−0.0820 (3)	0.0666 (2)	0.0759 (8)	
H29	0.1740	−0.0628	0.0531	0.091*	
C30	0.0760 (3)	−0.1537 (3)	0.1224 (2)	0.0828 (9)	
H30	0.1459	−0.1811	0.1459	0.099*	
C31	0.2674 (2)	0.1347 (2)	0.54507 (17)	0.0488 (5)	
H31	0.3170	0.2031	0.5897	0.059*	
C32	0.1434 (2)	0.1140 (2)	0.55870 (16)	0.0476 (5)	
H32	0.1111	0.1678	0.6114	0.057*	
C33	0.06698 (17)	0.01304 (17)	0.49382 (13)	0.0346 (4)	
C34	0.1222 (2)	−0.0622 (2)	0.41663 (16)	0.0470 (5)	
H34	0.0751	−0.1310	0.3704	0.056*	
C35	0.2468 (2)	−0.0349 (2)	0.40867 (17)	0.0503 (5)	
H35	0.2820	−0.0869	0.3567	0.060*	
N1	−0.33367 (14)	0.29750 (13)	0.10383 (11)	0.0338 (3)	
N2	−0.32931 (14)	0.50875 (13)	0.30290 (10)	0.0322 (3)	
N3	−0.0353 (3)	−0.18546 (18)	0.14443 (15)	0.0660 (6)	
N4	0.31901 (15)	0.06186 (16)	0.47140 (13)	0.0441 (4)	
O1	0.31950 (14)	0.38603 (18)	0.05648 (12)	0.0632 (5)	
O2	0.12403 (13)	0.42516 (15)	0.07502 (11)	0.0503 (4)	
O3	0.09298 (13)	0.54964 (12)	0.29372 (11)	0.0482 (4)	
O4	−0.06826 (11)	0.40345 (11)	0.22663 (9)	0.0348 (3)	
O5	0.07611 (12)	0.33003 (12)	0.39048 (9)	0.0390 (3)	
O6	−0.45077 (14)	0.00500 (14)	0.31796 (12)	0.0570 (4)	
O7	−0.45369 (14)	0.14505 (15)	0.46598 (12)	0.0591 (4)	
H7A	−0.5359	0.1073	0.4620	0.089*	
O8	−0.09546 (13)	0.65753 (11)	0.24523 (10)	0.0428 (3)	
H8B	−0.0773	0.6960	0.2039	0.064*	
H8A	−0.0193	0.6391	0.2712	0.064*	
O9	−0.13290 (12)	0.43834 (12)	0.03593 (9)	0.0394 (3)	
H9A	−0.0496	0.4192	0.0454	0.059*	
H9B	−0.1268	0.4886	0.0025	0.059*	
O10	−0.34951 (13)	0.57337 (13)	0.10870 (10)	0.0467 (4)	
H10A	−0.3992	0.6371	0.1457	0.070*	
H10B	−0.3349	0.5845	0.0581	0.070*	
O11	0.47994 (18)	0.25126 (16)	0.77447 (14)	0.0716 (5)	
H11B	0.5591	0.2710	0.7688	0.107*	
H11A	0.4599	0.1757	0.7487	0.107*	
O12	0.7365 (2)	0.32408 (19)	0.78175 (19)	0.0974 (7)	
H12A	0.7474	0.3571	0.7392	0.146*	
H12B	0.7518	0.3767	0.8394	0.146*	
Mn1	−0.21832 (2)	0.48542 (2)	0.172224 (18)	0.02771 (9)	

### Atomic displacement parameters (Å^2^)

**Table:**

	*U*^11^	*U*^22^	*U*^33^	*U*^12^	*U*^13^	*U*^23^
C1	0.0356 (10)	0.0460 (11)	0.0300 (9)	0.0099 (8)	0.0119 (7)	0.0183 (8)
C2	0.0266 (8)	0.0398 (10)	0.0286 (8)	0.0061 (7)	0.0056 (7)	0.0151 (7)
C3	0.0353 (10)	0.0514 (12)	0.0410 (10)	0.0162 (9)	0.0126 (8)	0.0198 (9)
C4	0.0471 (12)	0.0560 (13)	0.0590 (13)	0.0237 (10)	0.0119 (10)	0.0313 (11)
C5	0.0468 (12)	0.0570 (13)	0.0485 (11)	0.0110 (10)	0.0060 (9)	0.0354 (10)
C6	0.0276 (9)	0.0441 (10)	0.0303 (9)	−0.0006 (8)	0.0040 (7)	0.0178 (8)
C7	0.0232 (8)	0.0353 (9)	0.0258 (8)	0.0018 (7)	0.0028 (6)	0.0120 (7)
C8	0.0317 (9)	0.0367 (10)	0.0230 (8)	0.0058 (7)	0.0089 (7)	0.0121 (7)
C9	0.0309 (9)	0.0435 (10)	0.0289 (8)	0.0010 (8)	0.0043 (7)	0.0204 (8)
C10	0.0453 (11)	0.0454 (11)	0.0346 (10)	−0.0045 (9)	0.0110 (8)	0.0082 (8)
C11	0.0442 (11)	0.0477 (12)	0.0438 (11)	0.0029 (9)	0.0202 (9)	0.0137 (9)
C12	0.0340 (10)	0.0403 (10)	0.0401 (10)	0.0038 (8)	0.0058 (8)	0.0215 (8)
C13	0.0383 (10)	0.0393 (10)	0.0352 (10)	−0.0006 (8)	0.0011 (8)	0.0107 (8)
C14	0.0383 (10)	0.0473 (11)	0.0308 (9)	0.0042 (9)	0.0095 (8)	0.0124 (8)
C15	0.0349 (10)	0.0448 (11)	0.0529 (12)	0.0052 (9)	0.0066 (9)	0.0277 (10)
C16	0.0278 (9)	0.0636 (13)	0.0347 (10)	−0.0014 (9)	0.0081 (8)	0.0170 (9)
C17	0.0303 (10)	0.0649 (13)	0.0255 (9)	−0.0017 (9)	0.0054 (7)	0.0141 (9)
C18	0.0299 (9)	0.0355 (9)	0.0267 (8)	0.0017 (7)	0.0084 (7)	0.0103 (7)
C19	0.0274 (10)	0.0747 (15)	0.0362 (10)	0.0059 (9)	0.0084 (8)	0.0278 (10)
C20	0.0344 (10)	0.0663 (13)	0.0301 (9)	0.0061 (9)	0.0074 (8)	0.0232 (9)
C21	0.0447 (11)	0.0381 (10)	0.0356 (10)	−0.0010 (8)	−0.0047 (8)	0.0160 (8)
C22	0.0436 (11)	0.0337 (10)	0.0388 (10)	0.0023 (8)	−0.0007 (8)	0.0186 (8)
C23	0.0245 (8)	0.0324 (9)	0.0349 (9)	0.0049 (7)	0.0071 (7)	0.0129 (7)
C24	0.0513 (12)	0.0432 (11)	0.0331 (10)	−0.0078 (9)	−0.0046 (8)	0.0185 (8)
C25	0.0511 (12)	0.0425 (11)	0.0395 (10)	−0.0071 (9)	−0.0020 (9)	0.0234 (9)
C26	0.0784 (18)	0.0490 (13)	0.0538 (14)	−0.0004 (12)	0.0223 (13)	0.0144 (11)
C27	0.0596 (14)	0.0508 (13)	0.0529 (13)	0.0089 (11)	0.0139 (11)	0.0181 (10)
C28	0.0546 (13)	0.0408 (11)	0.0398 (10)	0.0057 (9)	0.0086 (9)	0.0119 (9)
C29	0.0595 (16)	0.091 (2)	0.100 (2)	0.0088 (15)	0.0103 (15)	0.0625 (18)
C30	0.080 (2)	0.091 (2)	0.099 (2)	0.0118 (17)	0.0021 (17)	0.0623 (19)
C31	0.0391 (11)	0.0498 (12)	0.0545 (13)	−0.0025 (9)	0.0011 (9)	0.0180 (10)
C32	0.0404 (11)	0.0472 (12)	0.0470 (11)	0.0034 (9)	0.0087 (9)	0.0062 (9)
C33	0.0323 (10)	0.0382 (10)	0.0359 (9)	0.0061 (8)	0.0036 (7)	0.0162 (8)
C34	0.0413 (11)	0.0472 (12)	0.0455 (11)	0.0034 (9)	0.0085 (9)	0.0075 (9)
C35	0.0452 (12)	0.0560 (13)	0.0531 (12)	0.0142 (10)	0.0201 (10)	0.0184 (11)
N1	0.0328 (8)	0.0338 (8)	0.0363 (8)	0.0010 (6)	0.0055 (6)	0.0150 (7)
N2	0.0312 (8)	0.0373 (8)	0.0299 (7)	0.0030 (6)	0.0108 (6)	0.0131 (6)
N3	0.0975 (18)	0.0476 (11)	0.0549 (12)	0.0008 (11)	0.0103 (12)	0.0225 (10)
N4	0.0341 (9)	0.0530 (10)	0.0556 (10)	0.0065 (8)	0.0080 (8)	0.0315 (9)
O1	0.0452 (9)	0.1113 (14)	0.0665 (10)	0.0290 (9)	0.0302 (8)	0.0637 (10)
O2	0.0394 (8)	0.0806 (11)	0.0559 (9)	0.0230 (7)	0.0185 (7)	0.0490 (8)
O3	0.0433 (8)	0.0358 (8)	0.0540 (9)	0.0023 (6)	0.0000 (7)	0.0039 (6)
O4	0.0253 (6)	0.0415 (7)	0.0448 (7)	0.0058 (5)	0.0076 (5)	0.0232 (6)
O5	0.0361 (7)	0.0532 (8)	0.0275 (6)	−0.0054 (6)	0.0077 (5)	0.0159 (6)
O6	0.0451 (9)	0.0530 (9)	0.0670 (10)	−0.0094 (7)	0.0011 (8)	0.0191 (8)
O7	0.0371 (8)	0.0688 (10)	0.0696 (10)	−0.0007 (7)	0.0192 (7)	0.0212 (8)
O8	0.0461 (8)	0.0319 (6)	0.0512 (7)	0.0025 (5)	0.0080 (6)	0.0159 (5)
O9	0.0374 (7)	0.0511 (8)	0.0389 (6)	0.0107 (6)	0.0165 (5)	0.0238 (5)
O10	0.0509 (9)	0.0626 (9)	0.0447 (8)	0.0262 (7)	0.0211 (6)	0.0336 (7)
O11	0.0669 (11)	0.0617 (11)	0.0820 (12)	0.0184 (9)	0.0249 (10)	0.0142 (9)
O12	0.0856 (15)	0.0685 (13)	0.1202 (18)	−0.0170 (11)	0.0203 (13)	0.0158 (12)
Mn1	0.02765 (15)	0.03050 (15)	0.02832 (15)	0.00391 (10)	0.00967 (10)	0.01315 (11)

### Geometric parameters (Å, º)

**Table:**

C1—O1	1.242 (2)	C23—C24	1.389 (3)
C1—O2	1.248 (2)	C23—C23^ii^	1.496 (3)
C1—C2	1.514 (2)	C24—C25	1.384 (3)
C2—C3	1.390 (3)	C24—H24	0.9300
C2—C7	1.402 (2)	C25—N1	1.335 (2)
C3—C4	1.380 (3)	C25—H25	0.9300
C3—H3	0.9300	C26—N3	1.317 (4)
C4—C5	1.388 (3)	C26—C27	1.382 (3)
C4—H4	0.9300	C26—H26	0.9300
C5—C6	1.371 (3)	C27—C28	1.387 (3)
C5—H5	0.9300	C27—H27	0.9300
C6—C7	1.388 (2)	C28—C29	1.379 (4)
C6—O5	1.404 (2)	C28—C28^iii^	1.495 (4)
C7—C8	1.518 (2)	C29—C30	1.380 (4)
C8—O3	1.250 (2)	C29—H29	0.9300
C8—O4	1.255 (2)	C30—N3	1.324 (4)
C9—O5	1.378 (2)	C30—H30	0.9300
C9—C14	1.380 (3)	C31—N4	1.325 (3)
C9—C10	1.384 (3)	C31—C32	1.381 (3)
C10—C11	1.378 (3)	C31—H31	0.9300
C10—H10	0.9300	C32—C33	1.384 (3)
C11—C12	1.387 (3)	C32—H32	0.9300
C11—H11	0.9300	C33—C34	1.389 (3)
C12—C13	1.386 (3)	C33—C33^iv^	1.488 (4)
C12—C15	1.487 (3)	C34—C35	1.377 (3)
C13—C14	1.387 (3)	C34—H34	0.9300
C13—H13	0.9300	C35—N4	1.324 (3)
C14—H14	0.9300	C35—H35	0.9300
C15—O6	1.218 (3)	N1—Mn1	2.2982 (15)
C15—O7	1.305 (3)	N2—Mn1	2.2852 (14)
C16—N2	1.332 (2)	O4—Mn1	2.1842 (12)
C16—C17	1.385 (3)	O7—H7A	0.9440
C16—H16	0.9300	O8—Mn1	2.2002 (13)
C17—C18	1.387 (3)	O8—H8B	0.8974
C17—H17	0.9300	O8—H8A	0.9473
C18—C19	1.391 (3)	O9—Mn1	2.1780 (12)
C18—C18^i^	1.493 (3)	O9—H9A	0.9544
C19—C20	1.378 (3)	O9—H9B	0.8941
C19—H19	0.9300	O10—Mn1	2.1543 (13)
C20—N2	1.336 (2)	O10—H10A	0.9941
C20—H20	0.9300	O10—H10B	0.8189
C21—N1	1.335 (2)	O11—H11B	0.8822
C21—C22	1.379 (3)	O11—H11A	0.8438
C21—H21	0.9300	O12—H12A	0.8519
C22—C23	1.386 (2)	O12—H12B	0.8467
C22—H22	0.9300		
			
O1—C1—O2	125.40 (17)	C23—C24—H24	120.0
O1—C1—C2	116.66 (16)	N1—C25—C24	123.56 (17)
O2—C1—C2	117.94 (15)	N1—C25—H25	118.2
C3—C2—C7	119.39 (16)	C24—C25—H25	118.2
C3—C2—C1	118.72 (16)	N3—C26—C27	124.1 (2)
C7—C2—C1	121.88 (15)	N3—C26—H26	117.9
C4—C3—C2	121.53 (18)	C27—C26—H26	117.9
C4—C3—H3	119.2	C26—C27—C28	119.6 (2)
C2—C3—H3	119.2	C26—C27—H27	120.2
C3—C4—C5	119.19 (18)	C28—C27—H27	120.2
C3—C4—H4	120.4	C29—C28—C27	116.0 (2)
C5—C4—H4	120.4	C29—C28—C28^iii^	122.3 (3)
C6—C5—C4	119.33 (18)	C27—C28—C28^iii^	121.7 (3)
C6—C5—H5	120.3	C28—C29—C30	120.3 (3)
C4—C5—H5	120.3	C28—C29—H29	119.9
C5—C6—C7	122.68 (17)	C30—C29—H29	119.9
C5—C6—O5	119.04 (16)	N3—C30—C29	123.5 (3)
C7—C6—O5	117.88 (16)	N3—C30—H30	118.2
C6—C7—C2	117.85 (16)	C29—C30—H30	118.2
C6—C7—C8	118.13 (15)	N4—C31—C32	123.0 (2)
C2—C7—C8	123.80 (15)	N4—C31—H31	118.5
O3—C8—O4	126.15 (16)	C32—C31—H31	118.5
O3—C8—C7	116.44 (15)	C31—C32—C33	119.97 (19)
O4—C8—C7	117.32 (15)	C31—C32—H32	120.0
O5—C9—C14	123.86 (16)	C33—C32—H32	120.0
O5—C9—C10	115.14 (16)	C32—C33—C34	116.35 (18)
C14—C9—C10	121.01 (17)	C32—C33—C33^iv^	121.7 (2)
C11—C10—C9	119.41 (18)	C34—C33—C33^iv^	122.0 (2)
C11—C10—H10	120.3	C35—C34—C33	119.9 (2)
C9—C10—H10	120.3	C35—C34—H34	120.1
C10—C11—C12	120.90 (18)	C33—C34—H34	120.1
C10—C11—H11	119.6	N4—C35—C34	123.2 (2)
C12—C11—H11	119.6	N4—C35—H35	118.4
C13—C12—C11	118.54 (17)	C34—C35—H35	118.4
C13—C12—C15	119.71 (18)	C25—N1—C21	116.29 (16)
C11—C12—C15	121.73 (18)	C25—N1—Mn1	123.23 (12)
C12—C13—C14	121.42 (18)	C21—N1—Mn1	120.44 (12)
C12—C13—H13	119.3	C16—N2—C20	116.29 (15)
C14—C13—H13	119.3	C16—N2—Mn1	120.71 (12)
C9—C14—C13	118.57 (17)	C20—N2—Mn1	122.44 (12)
C9—C14—H14	120.7	C26—N3—C30	116.5 (2)
C13—C14—H14	120.7	C35—N4—C31	117.55 (18)
O6—C15—O7	123.89 (19)	C8—O4—Mn1	130.26 (11)
O6—C15—C12	122.74 (19)	C9—O5—C6	119.08 (14)
O7—C15—C12	113.36 (18)	C15—O7—H7A	112.0
N2—C16—C17	123.57 (17)	Mn1—O8—H8B	113.1
N2—C16—H16	118.2	Mn1—O8—H8A	107.1
C17—C16—H16	118.2	H8B—O8—H8A	108.2
C16—C17—C18	120.29 (17)	Mn1—O9—H9A	111.3
C16—C17—H17	119.9	Mn1—O9—H9B	121.5
C18—C17—H17	119.9	H9A—O9—H9B	104.8
C17—C18—C19	115.83 (16)	Mn1—O10—H10A	126.1
C17—C18—C18^i^	121.8 (2)	Mn1—O10—H10B	118.1
C19—C18—C18^i^	122.4 (2)	H10A—O10—H10B	108.6
C20—C19—C18	120.22 (17)	H11B—O11—H11A	110.6
C20—C19—H19	119.9	H12A—O12—H12B	110.1
C18—C19—H19	119.9	O10—Mn1—O9	87.89 (5)
N2—C20—C19	123.78 (17)	O10—Mn1—O4	172.92 (5)
N2—C20—H20	118.1	O9—Mn1—O4	86.24 (5)
C19—C20—H20	118.1	O10—Mn1—O8	90.91 (6)
N1—C21—C22	123.81 (17)	O9—Mn1—O8	94.57 (5)
N1—C21—H21	118.1	O4—Mn1—O8	85.61 (5)
C22—C21—H21	118.1	O10—Mn1—N2	91.23 (5)
C21—C22—C23	120.05 (17)	O9—Mn1—N2	171.20 (5)
C21—C22—H22	120.0	O4—Mn1—N2	95.17 (5)
C23—C22—H22	120.0	O8—Mn1—N2	94.20 (5)
C22—C23—C24	116.28 (16)	O10—Mn1—N1	94.61 (6)
C22—C23—C23^ii^	121.71 (19)	O9—Mn1—N1	86.12 (5)
C24—C23—C23^ii^	122.0 (2)	O4—Mn1—N1	88.96 (5)
C25—C24—C23	119.98 (17)	O8—Mn1—N1	174.46 (5)
C25—C24—H24	120.0	N2—Mn1—N1	85.22 (5)

Symmetry codes: (i) −*x*−1, −*y*+1, −*z*+1; (ii) −*x*−1, −*y*, −*z*; (iii) −*x*, −*y*, −*z*; (iv) −*x*, −*y*, −*z*+1.

### Hydrogen-bond geometry (Å, º)

**Table:**

*D*—H···*A*	*D*—H	H···*A*	*D*···*A*	*D*—H···*A*
O7—H7*A*···N4^v^	0.94	1.64	2.577 (2)	170
O8—H8*B*···N3^vi^	0.90	1.94	2.814 (2)	166
O8—H8*A*···O3	0.95	1.76	2.662 (2)	158
O9—H9*A*···O2	0.95	1.87	2.8035 (19)	165
O9—H9*B*···O2^vii^	0.89	1.77	2.6595 (18)	174
O10—H10*A*···O11^viii^	0.99	1.78	2.771 (2)	173
O10—H10*B*···O1^vii^	0.82	1.84	2.6545 (19)	174
O11—H11*B*···O12	0.88	1.93	2.803 (3)	170
O11—H11*A*···O6^iv^	0.84	2.02	2.847 (2)	168
O12—H12*A*···O3^ix^	0.85	2.14	2.802 (3)	134
O12—H12*B*···O2^ix^	0.85	2.45	3.139 (3)	139

Symmetry codes: (iv) −*x*, −*y*, −*z*+1; (v) *x*−1, *y*, *z*; (vi) *x*, *y*+1, *z*; (vii) −*x*, −*y*+1, −*z*; (viii) −*x*, −*y*+1, −*z*+1; (ix) −*x*+1, −*y*+1, −*z*+1.
